# Supplementary material for: Yiai Fuzheng decoction inhibits triple-negative breast cancer by remodeling the immune microenvironment
Source: Front Immunol. 2025 Sep 30;16:1615631. doi: 10.3389/fimmu.2025.1615631 (PMC12518410; doi:10.3389/fimmu.2025.1615631)
Supplement: Supplementary file 2 [file DataSheet1.zip › Supplementary File 2/Treatment/C_vs_D/07_Pathway_Analysis/Pathway_Analysis/Sig_Pathways_ID/Galactose metabolism.pdf]

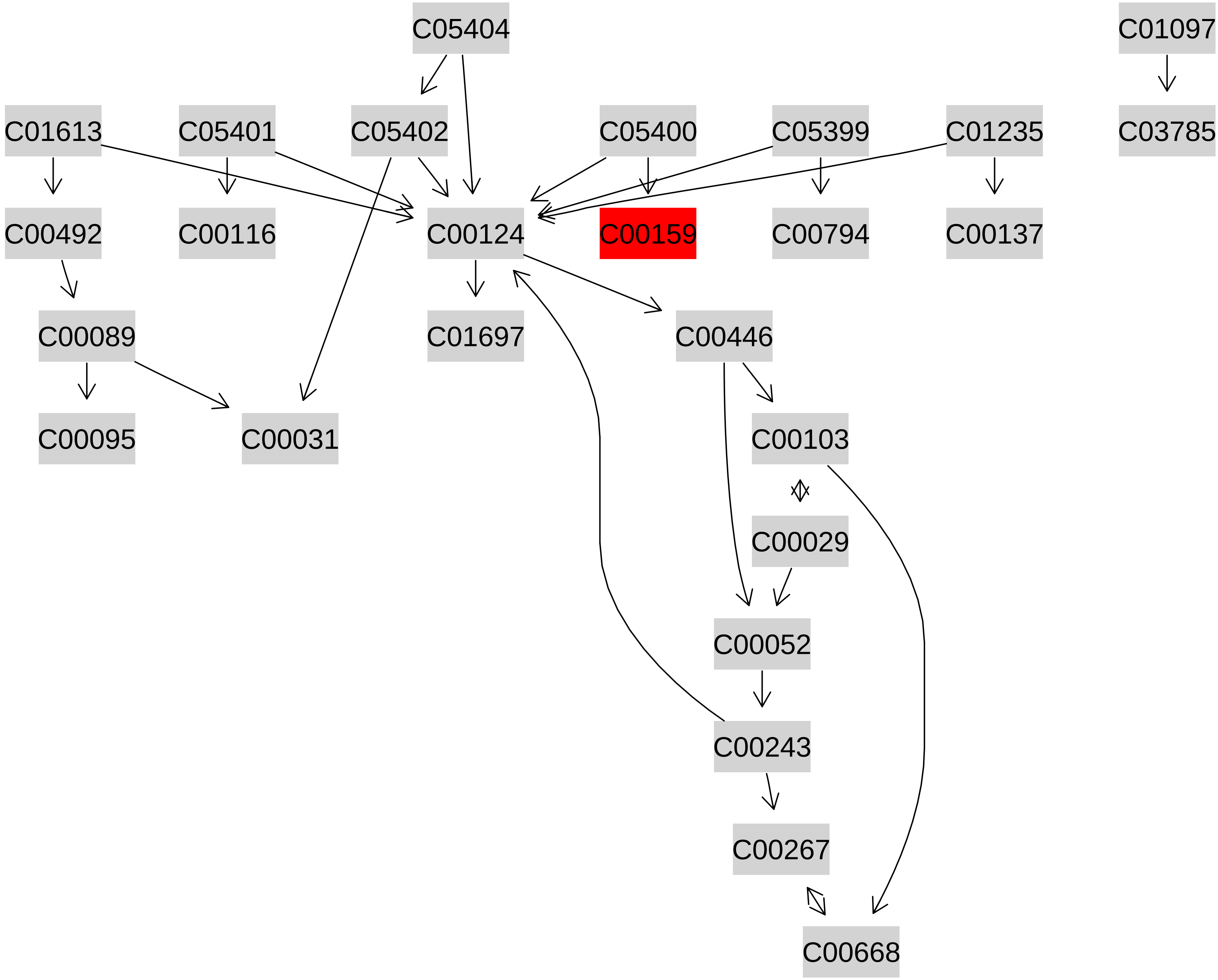
C05404
C01097
C01613
C05401
C05402
C05400
C05399
C01235
C03785
C00492
C00116
C00124
C00159
C00794
C00137
C00089
C01697
C00446
C00095
C00031
C00103
C00029
C00052
C00243
C00267
C00668
